# Exploring the relationship between ADHD, its common comorbidities, and their relationship to organizational skills

**DOI:** 10.7717/peerj.12836

**Published:** 2022-01-20

**Authors:** Guillaume Durand, Ioana-Smarandita Arbone

**Affiliations:** 1 Counselling, Psychotherapy, and Spirituality, St. Paul University, Ottawa, Canada; 2 University of Toronto, Toronto, Canada

**Keywords:** ADHD, Organizational skills, Sex differences, Depression, Comorbid disorders, Anxiety

## Abstract

**Background:**

Attention Deficit Hyperactivity Disorder (ADHD) is a neurodevelopmental disorder affecting numerous executive functioning skills, such as organizational skills. While the relationship between the inattention aspect of ADHD and poor organizational skills is well documented, it is still unclear if lower organizational skills are only associated with ADHD or if they are also associated with other comorbid disorders commonly diagnosed in conjunction with ADHD. The purpose of the present study is to investigate the relationship between organizational skills and ADHD in adults, as well as the impact of comorbid disorders on ADHD in relation to organizational skills.

**Methods:**

Four hundred seven (*n* = 201 with a diagnosis of ADHD) adults from the general population were recruited online. Participants completed a measure of organizational skills, measures assessing levels of ADHD, depression, and anxiety, and extensive demographic information related to their diagnosis of ADHD or other possible diagnosis.

**Results:**

Participants with a diagnosis of ADHD were significantly more likely to have a comorbid diagnosis of depression and/or anxiety. Organizational skills were significantly lower in individuals who reported having received a diagnosis of ADHD, but not in those with a past diagnosis of depression and anxiety. However, organizational skills were lower in individuals currently experiencing higher levels of depression and anxiety. The results of regression analyses suggest that levels of organizational skills are most strongly predicted by inattention and more weakly predicted by comorbid disorders.

**Discussion:**

These results highlight the centrality of organizational skills deficit in ADHD as well as the influence of the inattention component over other components commonly observed in ADHD on organizational skills. Suggestions for treatment of ADHD in adults are discussed.

## Introduction

The DSM-V lists attention deficit hyperactivity disorder (ADHD) as a neurodevelopmental disorder that causes significant impairments in the areas of attention, hyperactivity, and impulsivity. ADHD can be divided into three subtypes based on severity: inattentive presentation (ADHD-I), hyperactive-impulsive presentation (ADHD-HI), or combined presentation (ADHD-C; American Psychiatric Association, 2013). Worldwide, the prevalence of ADHD in adults 18 to 44 years old is between 1.1% and 7.3% (Fayyad et al., 2007).

### Significance of organizational skills for individuals with ADHD

Many organizational skills are challenging areas for individuals with ADHD which may have long-term impacts if inappropriately addressed. The inattention component may result in a decrease in the ability to plan and organize daily activities, which is a common drawback in some individuals with ADHD in adulthood (Kooij & Francken, 2010). Even when ADHD symptoms subside to subthreshold levels, the affected individuals still experience difficulties, particularly functional and educational impairments (Murphy et al., 2018). Additionally, good planning will likely help manage ADHD overall more effectively, including symptoms of  inquisitiveness or restlessness, which also pose significant challenges in adulthood (Kooij & Francken, 2010). 

Organizational skills may be particularly relevant in alleviating the negative impact of ADHD, given the link between ADHD and difficulty with executive functioning skills (Willcutt et al., 2005). Results suggest that 30% to 50% of individuals with ADHD experience a reduction in executive function as opposed to individuals without ADHD (Barkley & Murphy, 2010). Executive functions regulate behavior through higher-order thinking skills such as planning, organization, inhibition, and working memory. All of these functions enable an individual to regulate their behaviors and thinking in order to achieve a certain goal (Baddeley & Hitch, 1994).

Executive functions seem to be commonly impaired in children with ADHD, even when the symptoms of inattention are not particularly severe (Baddeley & Hitch, 1974; Willcutt et al., 2005). However, while some children and teenagers will experience more inattention and less of a reduction in executive functions, other children and teenagers may experience the opposite (Biederman et al., 2007). Furthermore, difficulties with executive functions often become more influential as the individual ages due to decreased external structures (Evans, Green & Serpell, 2005; Evans et al., 2005), even though symptoms of impulsivity and hyperactivity are likely to subside with age (Biederman, Mick & Faraone, 2000).

### ADHD comorbidities’ negative impact on ADHD symptomatology and organizational skills

The challenges with organizational skills are compounded by the ADHD’s rates of comorbidity. Indeed, over 75% of individuals with ADHD experience at least one other psychiatric disorder (Sobanski et al., 2007). ADHD is also far more prevalent in psychiatric populations, with estimates from 2-fold to 7-fold (Kessler et al., 2006). The types of comorbidities tend to vary based on ADHD type, with ADHD-I and ADHD-C exhibiting higher rates of internalizing problems (Lahey & Willcutt, 2010) and ADHD-HI presenting with higher rates of externalizing disorders (Diamond, 2005). Nonetheless*,* some of the most common disorders associated with ADHD include depression and anxiety.

Internalizing disorders such as depression and anxiety are notably common among people with ADHD. Depression is alleged to be about five times as common among individuals with ADHD than among those without it (Angold, Costello & Erkanli, 1999). The co-occurrence of severity of ADHD and depression has long been assumed to be the result of demoralization arising from life struggles caused by untreated ADHD in children and adults (Biederman, Mick & Faraone, 1998; Herman et al., 2007). Previous studies suggest that not only is depression associated with lower cognitive functioning, but these depressive symptoms partly explain the association between ADHD and cognitive abilities (Semeijn et al., 2015).

Anxiety is commonplace in around 25% of individuals with ADHD (Jarrett & Ollendick, 2008) though there is significant variance in ADHD rates (Busch et al., 2002; Jensen et al., 2001b; Jensen et al., 2001a). There is evidence that anxiety may negatively affect working memory among individuals with ADHD and that comorbid profiles are characterized by more negative affectivity and social problems (Jensen et al., 2001a; Jensen et al., 2001b; Levy, 2004). There may, however, be a positive relationship between anxiety and impulsivity, since in some individuals impulsive behavior may be overridden by anxiety (Jarrett & Ollendick, 2008).

### Biological sex and age influences on ADHD and organizational skills

Furthermore, ADHD may have differential impacts depending on the sex of the person affected. ADHD is about 1.6 times more common in men than in women (Willcutt, 2012), and it is not clear whether the female and male ADHD profile is different (Biederman et al., 2004).  It is suspected that professionals are more likely to diagnose men with this disorder, regardless of the symptom presentation (Bruchmüller, Margraf & Schneider, 2012). Nevertheless, there is evidence that sex may be a contributing factor, affecting symptomatology. Skogli et al. (2013) reported that higher anxiety is associated with women affected by ADHD, whereas rule-breaking was cited more often for boys. Though diagnostic practices and symptomatology may differ by sex, neuropsychological tests do not seem to substantially differentiate between men and women with ADHD (Skogli et al., 2013). At the same time, sex may be an important factor to consider when developing treatment for ADHD. Indeed, women may have a better reaction to ADHD remediation strategies that are specific to symptoms whereas men might be more receptive when the intervention involves social interaction (Elkins et al., 2018).

A second factor that needs to be considered is age. Kooij & Francken (2010) state that ADHD is often under-diagnosed in adults because of: lack of knowledge about the disorder, differences in symptom presentations with age, and the presence of comorbidities. However, adults have been found to be given accurate reports of their ADHD childhood symptoms (Murphy & Schachar, 2000). To have a diagnosis of ADHD, it is required for symptoms to have been present since childhood. The age of ADHD onset in childhood, whether early (between 0 and 7 years old) or late (between 7 and 12 years old), is associated with similar comorbidities (Lin et al., 2015). However, there are notable interactions between ADHD symptoms and other comorbidities. For example, a recent study found that presence of externalizing behaviors in middle childhood predicts ADHD in adolescence, while ADHD in adolescence predicts externalizing behaviors in adulthood (Kuja-Halkola et al., 2015). This recent study goes against the assumption that externalizing behavior disorders occur because of ADHD. Rates of adulthood ADHD are reported at around 2–4% (Adler et al., 2009; Biederman, 2005; Kessler et al., 2006). 

### Characteristics related to ADHD impacting organizational skills

In addition to sex and age, other characteristics may moderate the relationship between executive functioning, such as organizational skills, and ADHD. For instance, previous studies suggested that individuals diagnosed with ADHD receiving medication performed better on a series of cognitive tasks as opposed to those with ADHD without medication (Kempton et al., 1999; Kessler et al., 2007). Past studies also suggest that psychotherapy, such as cognitive behavioral therapy, can improve executive functions in individuals with ADHD (Kessler et al., 2007).

The relationship between ADHD and other disorders and their combined impact on organizational skills remain unclear. The purpose of this study is to explore the relationship between organizational skills and ADHD, as well as disorders commonly comorbid with ADHD (*e.g.*, anxiety and depression). We have developed three hypotheses: (1) individuals diagnosed with ADHD will display higher levels of comorbid disorders, such as anxiety and depression; (2) organizational skills will be significantly lower in individuals with a diagnosis or high symptoms of anxiety, depression, or ADHD, with the largest effect size for the ADHD group; and (3) an exploratory regression analysis will examine the influence of comorbid disorders and common characteristics (age, sex, medication, therapy) by diagnosis of ADHD on organizational skills.

### Methods

### Participants

A total of four hundred and seven (*N* = 407) participants were recruited online on Facebook groups dedicated to individuals with ADHD and forums related to psychological research (*e.g.*, reddit/r/SampleSize). Inclusion criteria were to be over 18 years old and be fluent in English. There were no exclusion criteria. Participants were 24% male and 76% females. A total of 201 participants (49% of the sample) reported having received an official diagnosis of ADHD from a physician, a psychiatrist, or a psychologist. Participants were mostly native English speakers (81%). Regarding education, participants had either high school or college education (31%), bachelor education (39%), or post-bachelor education (29%). Most participants were in North America (65%) or Europe (25%). Almost half (42%) of the sample reported being currently enrolled as a student in a university. The mean age was 28.40 years old (*SD* = 9.21). An ANOVA suggests a mean age difference between groups, whereas individuals without an ADHD diagnosis (*M* = 26.79, *SD* = 8.57) were younger than those with an ADHD diagnosis (*M* = 30.04, *SD* = 9.56) (*F*(1, 405) = 13.127, *p* < .001, *d* = 0.36). All participants provided written informed consent prior beginning the online study. Participants were not compensated for participating in the study. The present study was approved and given ‘exempt’ status by the IntegReview Ethical Review Board (Austin, TX, USA), under protocol number 11022016.

### Measures

**Durand Organizational Skills Questionnaire (DOSQ; Durand, 2020).** The DOSQ is a self-reported instrument including 38 items distributed on eight factors assessing one’s organizational skills. In addition to a total score, the DOSQ provides subscales’ scores for core organizational skills: work organization, communication clarity, punctuality, goal-oriented behavior, assiduity, workspace organization, strategies, and attentiveness. Items are rated on a 6-point rating scale ranging from Strongly Disagree to Strongly Agree. Items include statements such as “*I rarely leave tasks to the last minute*“ and “*My friends and family would consider me a punctual person*“. A previous study assessing the relationship between the DOSQ and ADHD reported adequate internal consistency on the DOSQ’s total score (α = .86) and on its subscales (α = .60 to .95), as well as adequate construct validity with ADHD symptoms for adults (Durand, Arbone & Wharton, 2020).

**Adult ADHD Self-Report Scale (ASRS; Kessler et al., 2005).** The ASRS is an 18-item self-reported instrument assessing the presence of ADHD symptoms in adults. The items are rated on a 5-point scale (1 = never, 5 = very often). The scale is divided in two sections: Inattention (6 items, commonly used for screening) and Hyperactivity-Impulsivity (12 items, commonly used for additional cues). Past research has suggest a cut-off score of 20 (14 when using a 0 to 4 rating scale) to identify individuals with potential ADHD (Kessler et al., 2007). For this study, we used the cut-off score of ≥ 20 to identify individuals with high levels of ADHD symptoms. The instrument is commonly used in clinical settings and has demonstrated good psychometric properties (Kessler et al., 2007).

**Patient Health Questionnaire (PHQ-9; Kroenke, Spitzer & Williams, 2001).** The PHQ-9 is a self-report instrument measuring the severity of depressive symptoms based on the DSM-IV criteria for major depressive disorder. Nine items are rated on a 4-point scale (0 = not at all, 3 = nearly every day). A higher score reflects higher levels of depressive symptoms. For this study, we used the cut-off score of ≥ 15 to identify individuals with moderately severe to severe depressive symptoms within the last two weeks. The PHQ-9 is commonly used and possesses good psychometric properties of the PHQ-9 (Bronchain, Chabrol & Raynal, 2021).

**State-Trait Anxiety Inventory Short Form (STAI-6; Marteau & Bekker, 1992).** The STAI-6 is a 6-item version of the original 20-item state scale of the STAI (Spielberger, Gorsuch & Lushene, 1970). Items are rated on a 4-point scale (1 = not at all, 4 = very much). A higher score reflects higher levels of anxiety in the present. Although the STAI-6 should not be used for clinical diagnosis, we identify two groups within the sample: individuals scoring 1 standard deviation below the mean (low anxiety) and individuals scoring 1 standard deviation above the mean (high anxiety). Multiple studies support the good psychometric properties of the STAI-6 (Balta et al., 2020).

## Results

All analyses were performed by condition (no-ADHD/ADHD). The attribution to one group or the other (no-ADHD/ADHD) was based on the participant’s response to having received (or not) a diagnosis of ADHD from a health professional (physician, psychiatrist, or psychologist). Examination of Kurtosis and Skewness support a normal distribution on the DOSQ and its subscales, alongside the ASRS, the PHQ, and the STAI-6. Table 1 shows the descriptive data (mean, standard deviation, assumptions of normality and Cronbach’s alpha) for both conditions, as well as the Cohen’s *d* effect size on each scale between condition for all self-reported instruments in the present study. A series of analysis of variance (ANOVA) supported mean differences between conditions on the DOSQ Total (*F*(1, 405) = 50.638, *p* < .001), alongside most of its subscales: Work Organization (*F*(1, 405) = 23.474, *p* < .001), Communication Clarity (*F*(1, 405) = 28.208, *p* < .001), Punctuality (*F*(1, 405) = 31.946, *p* < .001), Assiduity (*F*(1, 405) = 103.410, *p* < .001), and Attentiveness (*F*(1, 405) = 103.464, *p* < .001). Differences were also observed on both ASRS scales, namely Inattention (*F*(1, 405) = 140.400, *p* < .001) and Hyperactivity-Impulsivity (*F*(1, 405) = 92.451, *p* < .001). Table 2 provides additional descriptive data specifically related to individuals with a diagnosis of ADHD in terms of taking medication, time since the diagnosis, and seeking therapy.

**Table 1:** Descriptive data.

	No ADHD diagnosis (*n* = 206)				ADHD diagnosis (*n* = 201)					
	Mean (SD)	α	β2	Skp	Mean (SD)	α	β2	Skp	*d*	*C.I. 95%*
DOSQ										
Total^**^	134.26 (34.76)	.95	−0.74	0.07	113.68 (22.02)	0.86	0.10	0.13	0.70	[0.50–0.90]
Work^**^	11.13 (3.63)	.71	−0.62	−0.26	9.44 (3.38)	0.65	−0.27	0.43	0.48	[0.28–0.68]
Communication^**^	22.67 (7.89)	.95	−0.87	−0.17	18.71 (7.11)	0.93	−0.39	0.34	0.53	[0.33–0.72]
Punctuality^**^	12.57 (4.56)	.96	−0.76	−0.64	9.91 (4.92)	0.95	−1.20	0.19	0.56	[0.36–0.76]
Goal-Oriented	12.83 (5.41)	.86	−0.89	0.06	12.22 (4.82)	0.79	−0.68	0.21	0.12	[−0.08–0.31]
Assiduity^**^	18.34 (7.09)	.87	−0.86	0.28	12.31 (4.56)	0.73	1.36	0.99	1.01	[0.80–1.21]
Workspace	18.89 (5.71)	.85	−0.53	−0.14	18.05 (4.99)	0.77	−0.28	−0.02	0.16	[−0.04–0.35]
Strategies	23.60 (8.75)	.88	−0.83	0.12	23.48 (7.85)	0.83	−0.78	0.03	0.01	[−0.18–0.21]
Attentiveness^**^	14.23 (5.32)	.84	−1.00	−0.03	9.54 (3.85)	0.68	0.50	0.82	1.01	[0.80–1.21]
ASRS										
Inattention^**^	28.94 (7.13)	.85	−0.43	0.02	36.19 (5.01)	0.76	0.28	−0.60	1.18	[−1.38 to −0.96]
Hyperactivity^**^	25.04 (6.19)	.78	−0.17	0.34	30.90 (6.08)	0.79	−0.17	−0.32	0.96	[−1.16 to −0.75]
PHQ										
Total	11.09 (6.83)	.88	−0.83	0.34	12.18 (6.28)	0.85	−0.73	0.25	0.17	[−0.36–0.03]
STAI-6										
Total	14.35 (4.39)	.86	−0.91	−0.06	14.96 (4.05)	0.84	−0.59	0.12	0.14	[−0.34–0.05]

**Notes.**

* *p* < .01. ** *p* < .001 indicates a significant diagnosis difference. β2, Kurtosis; Skp, Skewness; Work, Work Organization; Communication, Communication Clarity; Goal, Goal-Oriented Behavior; Workspace, Workspace Organization; Hyperactivity, Hyperactivity-Impulsivity.

**Table 2:** DOSQ total score by characteristics associated with the ADHD diagnosis in participants with an ADHD diagnosis (*n* = 201).

	*N*	*Mean*	*SD*
Are you taking medication?			
Yes	134	112.87	21.26
No, and I never took any	18	113.50	21.72
I used to, but not anymore	49	115.96	24.37
When were you diagnosed with ADHD?			
Less than a year ago	47	111.51	18.66
More than 1, less than 3	42	107.69	21.07
More than 3, less than 5	15	116.53	23.84
Between 5 and 10	37	118.08	27.17
More than 10 years ago	60	116.13	20.71
Are you seeking therapy for ADHD?			
Yes	43	112.42	21.22
No	116	113.32	22.42
I used to, but it did not help	28	111.54	21.95
I used to, and it helped	14	124.78	20.23

Chi-square tests were performed between groups on six independent variables, namely education, biological sex, depression level, anxiety level, ADHD level, and comorbid diagnoses, and are reported in Table 3. For comorbid diagnoses, participants answered the question “Do you have (or have you received in the past) a diagnosis for […] from a physician, a psychiatrist, or a psychologist?”. Overall, individuals with a diagnosis of ADHD had a higher likelihood of being female and having a diagnosis of depression or anxiety, as well as having higher levels of self-reported ADHD symptoms. However, the analysis did not support a difference between the groups in terms of the intensity of self-reported depressive symptoms and anxiety. Individuals without ADHD had a higher likelihood of never having received a diagnosis for a mental illness.

**Table 3:** Proportions of education levels, biological sex, and presence of a diagnosis within the No ADHD diagnosis and ADHD diagnosis groups.

	No ADHD diagnosis (*n* = 206)	ADHD diagnosis (*n* = 201)		
	N (%)	N (%)	*χ* ^2^	*p*
Education			6.92	.031
High school	52 (25%)	73 (38%)		
Bachelor	86 (42%)	71 (37%)		
Post-bachelor	66 (32%)	50 (26%)		
Biological sex			18.20	.000
Male	68 (33%)	30 (15%)		
Female	138 (67%)	171 (85%)		
Depression (PHQ)			1.77	.208
Low	143 (69%)	127 (63%)		
High	63 (31%)	74 (37%)		
Anxiety (STAI-6)			0.58	.487
Low	101 (49%)	91 (45%)		
High	105 (51%)	110 (55%)		
ADHD (ASRS)			65.78	.000
Low	112 (54%)	32 (16%)		
High	94 (46%)	169 (84%)		
Diagnoses				
Depression	83 (40%)	132 (66%)	26.29	.000
Anxiety	79 (38%)	127 (63%)	25.10	.000
Other	28 (14%)	40 (20%)	2.84	.092
No diagnosis	97 (47%)	32 (16%)	46.15	.000

**Notes.**

High school = High school and college (1–2 years programs). Depression Low refers to individuals with a score inferior to 15. Depression High refers to individuals with a score equal or superior to 15. Anxiety Low refers to individuals under the mean. Anxiety High refers to individuals above the mean. ADHD low refers to individuals with a score inferior to 20. ADHD high refers to individuals with a score equal or superior to 20. Percentages refers to ratio within groups. For example, 25% of individuals with no ADHD diagnosis have a high school degree. For diagnoses, percentages refer to the presence of a disorder within the group. For example, 40% of individuals with no ADHD diagnosis have a diagnosis of depression.

Subsequently, we examined the difference in organizational skills in terms of diagnoses and self-reported information related to ADHD, depression, and anxiety. To correct for multiple testing, a Bonferroni correction of *p* = .05/6 = .008 was applied to account for the six analyses. A series of ANOVAs confirmed that lower levels of organizational skills were present in individuals who received a diagnosis of ADHD (as mentioned in Table 1), but not in individuals who received a diagnosis (at some point in their past) of depression (*F*(1, 405) = 5.524, *p* = .019, *d* = 0.23) or anxiety (*F*(1, 405) = 6.860, *p* = .009, *d* = 0.26). Furthermore, lower levels of organizational skills were observed in individuals passing the threshold value on the ASRS (*F*(1, 405) = 201.535, *p* < .001, *d* = 1.48) and the PHQ (*F*(1, 405) = 25.053, *p* < .001, *d* = 0.52), as well as those above the mean on the STAI-6 (*F*(1, 405) = 22.785, *p* < .001, *d* = 0.47).

Lastly, we performed two regression analysis, one per group of ADHD diagnosis, to identify other variables measuring organizational skills. For individuals with a diagnosis of ADHD, a stepwise regression model was computed with the following variables: depression diagnosis, anxiety diagnosis, no diagnosis, sex, age, PHQ total, ASRS inattention total, ASRS hyper-impulsivity total, STAI-6 total, medication, and therapy. The results suggested a significant model (*F*(3, 197) = 38.324, *p* < .001, *adjusted R^2^* = .36). The only significant predictors were ASRS inattention (*Standardized B* = −0.532, *t* =  − 8.552, *p* < .001), ASRS hyper-impulsivity (*Standardized B* = 0.158, *t* = 2.548, *p* < .001) and self-reported depression total (*Standardized B* = −0.263, *t* =  − 4.380, *p* < .001). The first model, containing only inattention, explained 29% of the variance, increasing to 34% when also taking into account self-reported depression and 36% when also including hyperactivity-impulsivity.

A second regression analysis was performed for individuals without a diagnosis of ADHD with the aforementioned predictors, aside of medication and therapy. The results suggested a significant model (*F*(5, 199) = 88.503, *p* < .001, *adjusted R^2^* = .68). The significant predictors included sex (*Standardized B* = 0.145, *t* = 3.582, *p* < .001), anxiety diagnosis (*Standardized B* = 0.145, *t* = 3.445, *p* = .001), ASRS inattention (*Standardized B* = −0.818, *t* =  − 16.108, *p* < .001), ASRS hyper-impulsivity (*Standardized B* = 0.131, *t* = 2.842, *p* = .005), and self-reported depression total (*Standardized B* = −0.138, *t* =  − 2.888, *p* = .004). The first model, containing only inattention, explained 62% of the variance. The variance explained increased to 65% when also including sex, 66% when adding anxiety diagnosis, 67% when including self-reported depressive symptoms, and 68% when adding hyperactivity-impulsivity.

## Discussion

This study examined the relationship between ADHD and comorbid disorders, as well as their relationship with organizational skills. As expected, the results suggest that people with ADHD are more likely to have a comorbid diagnosis of depression (*p* < .001) or anxiety (*p* < .001). This finding is aligned with the literature, as previous results suggest that individuals with ADHD are five time more likely to have a diagnosis of depression than individuals without ADHD (Angold, Costello & Erkanli, 1999). Although associations between anxiety and ADHD vary considerably (Busch et al., 2002; Jensen et al., 2001a; Jensen et al., 2001b), findings suggest that 20% of individuals with ADHD are diagnosed with anxiety (Jarrett & Ollendick, 2008). Furthermore, the present study highlights a difference in psychiatric symptoms among the groups with/without ADHD, as half of the people with no ADHD reported having never received a diagnosis for a mental health issue, as opposed to only 16% for those with ADHD (excluding ADHD itself) (*p* < .001).

Interestingly, no significant difference was found when comparing groups of low and high levels of depression and anxiety. The discrepancy could be caused by the timeframe of the symptoms. For the diagnosis, individuals may have a current depression/anxiety disorder, or may have had one in the past that was eventually treated. However, self-reported instruments were referring to current (within the past few weeks) symptoms associated with depression and anxiety. The similar ratio of self-reported anxiety and depression in individuals with and without ADHD could be due to the large proportion of students in the sample size. Multiple studies suggest that university students display high levels of depressive and anxiety-related symptoms (Bayram & Bilgel, 2008; Eisenberg, Golberstein & Hefner, 2007). As expected, individuals with a diagnosis of ADHD were more likely to obtain a high score on the ASRS, while individuals without a diagnosis were more evenly distributed between low and high scores on the ASRS. There are two potential explanations for these results. First, the study was advertised as a study focusing on ADHD. It is possible that individuals who suspect they have ADHD may have been disproportionately more interested in participating in this study. Secondly, multiple studies suggest that many adults are not properly diagnosed once they reach adulthood, and that the majority of those undiagnosed individuals remain untreated (Montejano et al., 2011).

Our findings also suggest that individuals with a diagnosis of ADHD, as well as individuals with higher levels of self-reported depression, anxiety, and stress, display lower levels of organizational skills. Interestingly, this difference was not found for individuals with a current or past diagnosis of depression or anxiety disorder. As previously mentioned, these results may be due to the focus of self-reported instruments on current symptoms. Multiple studies suggest that anxiety and depression negatively impact executive functions concurrently (O’Rourke EJ & Vaysman, 2020; Rabin, Fogel & Nutter-Upham, 2011) or years in the future (Zainal & Newman, 2022). It is possible that individuals who currently experience symptoms associated to depression and anxiety experience a decline in executive functions, such as organizational skills. It is however unknown if there is a cause and effect or if it is simply a relationship. It is also currently unknown if these lower levels of organizational skills are temporary or permanent. Nevertheless, these results suggest that while common comorbid diagnosis of ADHD may play a role in reduced organizational skills, their contribution may not be as impactful as ADHD, considering the large difference in effect sizes between ADHD and self-reported anxiety and depression in relation to organizational skills.

In the present study, two regression analyses were computed to examine the significant predictors of organizational skills. The results suggested that, for both individuals with and without ADHD, the inattention component of the ASRS was the strongest predictor to measure organizational skills, assessing 29% and 62% of the variance for individuals with and without ADHD, respectively. Additional predictors, while statistically significant, were marginal, increasing the total variance by only 7% and 6%, respectively. These results confirm the centrality of the inattention factor in a reduction of organizational skills (Bikic et al., 2017). It is possible that, while other factors may impact the severity of ADHD as a disorder (such as comorbid disorders, sex, etc.), the inattention component has the strongest impact on reduced executive functioning, such as organizational skills. Future studies should explore the difference in individuals with and without the hyperactivity-impulsivity component of ADHD in order to further explore the role of the inattention component on organizational skills.

## Conclusion

The findings of this study suggest that organizational skills are a core facet of ADHD, weakly related to other comorbid disorders the individual with ADHD may be experiencing. The findings support the study’s hypotheses: individuals with ADHD are more likely to have or have received at some point a diagnosis of anxiety disorder or depression, organizational skills are lower in individuals reporting higher levels of symptoms of ADHD, depression, and anxiety, and regression analyses suggest that inattention is the largest predictor of organizational skills in individuals with and without ADHD.

Future studies should also explore the utility of therapies focused on increasing organizational skills in individuals with ADHD. The development of such interventions is particularly important, given that problems with organizational difficulties are likely to persist even when other ADHD symptoms lessen (Murphy et al., 2018), and given the contribution of organizational skills to functional and educational success (Barkley, 2014; Cantwell & Baker, 1991; Frick et al., 1991; Pastor & Reuben, 2002). Despite these findings, the present study has a few limitations, including a lack of employment-related information (managerial positions, success, job satisfaction), and a relatively small sample (*N* = 407) which may have prevented finding cases with rare diseases (*e.g.*, other common comorbid disorders to ADHD such as sleep disorders). It was also noted that the current study was possibly too long for those individuals with very severe ADHD. Therefore, it is recommended that future studies be shorter or be delivered in multi-steps. Additionally, the present study did not distinguish between current and past diagnosis of depression and anxiety. While a lack of distinction is less problematic for life-long pathologies (such as bipolar disorder), it is highly possible that some individuals were diagnosed in the past for depression or anxiety but are not currently experiencing symptoms. Future studies should take these cases into account when assessing comorbid disorders. Lastly, the participants included in the sample may not be an adequate representation of the general population. A large proportion of the sample included females with high levels of education. This could be due to some of the recruitment method, which focused on online support groups for ADHD. Future studies should attempt to recruit from the general population while keeping a better ratio between males and females.

## Supplemental Information

10.7717/peerj.12836/supp-1 Supplemental Information 1 Recoded data set

## Additional Information and Declarations

### Data Availability

The following information was supplied regarding data availability: The raw data is available in the Supplemental File.
